# Expanding the Developmental Origins of Health and Disease beyond maternal behaviors

**DOI:** 10.1371/journal.pmed.1005208

**Published:** 2026-07-30

**Authors:** Alice Rhiannon Lee, Daniel B. Hawcutt, Jennifer K. Quint, Ian P. Sinha

**Affiliations:** 1 Department of Research and Innovation, Alder Hey Children’s Hospital, Liverpool, United Kingdom; 2 Institute of Life Course and Medical Sciences, University of Liverpool, Liverpool, United Kingdom; 3 School of Public Health, Imperial College London, London, United Kingdom

## Abstract

Developmental Origins of Health and Disease (DOHaD) research has long emphasized maternal pregnancy behaviors as key determinants of child health. In this Perspective, Alice Rhiannon Lee and colleagues discuss new evidence that suggests the greatest opportunities to improve child health may lie in tackling socioeconomic disadvantage rather than maternal behavior alone.

## Teaser

Developmental Origins of Health and Disease (DOHaD) research has long emphasized maternal pregnancy behaviors as key determinants of child health. New evidence recently published in *PLOS Medicine* suggests the greatest opportunities to improve child health may lie in tackling socioeconomic disadvantage rather than maternal behavior alone.

Since its emergence in the late 20th century, the Developmental Origins of Health and Disease (DOHaD) paradigm has transformed understanding of how early-life exposures shape health across the life-course. In practice, DOHaD research and policy have focused overwhelmingly on maternal pregnancy exposures, reinforcing the implicit assumption that developmental influences are primarily mediated through mothers [1]. In a recent study in *PLOS Medicine* [2], Sharp and colleagues prompt us to reconsider this central assumption in DOHaD literature: are early-life influences on child health primarily a consequence of maternal behaviors during pregnancy, or do they instead reflect the broader constellation of influences that surround mothers, including partners and their wider socioeconomic environment?

Using triangulated data from four birth cohorts spanning two decades, their study (“Exploring Prenatal influences on Childhood Health” (EPoCH)) examined maternal and co-parent smoking, alcohol consumption, caffeine intake and socioeconomic position in relation to more than 70 child health outcomes across early childhood. By combining conventional epidemiological meta-analyses with mendelian randomization, negative control analyses, and genetic risk scores, they move beyond the observational associations that have underpinned much of DOHaD literature towards stronger causal inference of intrauterine effects from shared familial and wider socioeconomic influences.

Their findings indicate that these parental behaviors do not have large causal effects on childhood outcomes. Maternal smoking was the primary exception, remaining associated with small-for-gestational-age birth, later overweight and obesity, and six psychosocial outcomes. However, the associations between maternal smoking and body mass index were attenuated after adjustment for genetic risk scores, suggesting that the observed associations may be partly explained by shared genetic influences. In contrast, socioeconomic position showed the most consistent associations across outcomes, suggesting that broader social circumstances may exert a greater influence on child health than many individual prenatal behaviors.

By utilizing multiple birth cohorts and applying several complementary approaches to causal inference, Sharp and colleagues provide a comprehensive assessment of the relative contributions of modifiable behaviors and socioeconomic circumstances to child health. This combination of breadth and methodological triangulation represents an important contribution to DOHaD research. However, the predominance of white participants in the study limits assessment of how socioeconomic disadvantage intersects with ethnicity, an important consideration given persistent ethnic inequality in pregnancy outcomes in the UK [3].

The authors should be commended for their commitment to open science. By making their analytical framework and interactive EPoCH platform publicly available, they enable other researchers to interrogate the data, reproduce analyses, and generate new hypotheses. This collaborative approach represents an important model for the future of epidemiological research. The next challenge for DOHaD is not only to continue identifying the origins of health and disease, but to use these increasingly rich and expansive data to develop interventions that address the social and structural drivers of child health, rather than maternal behavior alone.

Perhaps the most important contribution of this work lies not in its methodological innovation, but in the questions it raises about where responsibility for child health should lie. The findings suggest that, for many outcomes, the contribution of maternal behaviors is modest compared with the pervasive influence of socioeconomic disadvantage.

This challenges the longstanding emphasis within DOHaD research and maternity care on modifying maternal behaviors during pregnancy, reflecting the assumption that these represent the principal opportunities to improve child health. Instead, the findings align with the wider literature on the social determinants of health, which shows that individual behaviors are shaped by the social and economic conditions in which people live. For example, an earlier study showed that persistent childhood poverty in Denmark is associated with increased all-cause mortality in early adulthood, highlighting the cumulative consequences of disadvantage across the life-course [4]. At the same time, evidence from diverse settings indicates that unconditional cash transfers during pregnancy can improve infant outcomes, demonstrating that upstream social interventions have measurable benefits for early-life health [5].

Socioeconomic disadvantage influences maternal and child health by shaping nutrition, housing quality, financial security, chronic stress, environmental exposures, access to healthcare and opportunities for healthy behaviors. The challenge is therefore not simply encouraging healthier choices but creating circumstances in which those choices are genuinely available. The proposed 3Cs framework of Clock, Cost and Capacity [6] provides a useful way of understanding these barriers, recognizing that families may lack the time, financial resources or physical and emotional capacity needed to act on health advice, irrespective of their motivation. Public health measures that promote healthy parental behaviors remain important, but they are likely to have limited impact, or even widen health inequalities, when they fail to address the structural and societal barriers that constrain families’ ability to act on this advice. For example, while the NHS Eatwell Guide provides freely available dietary guidance, the 2026 *Broken Plate* report estimated that households with children in the lowest income quintile would need to spend 85% of their disposable income to afford a healthy diet [7]. Combined with domestic energy costs, which accounted for a further 16.4% of disposable income in England in 2024 [8], the financial reality becomes clear: for many families, so-called “individual choices” are neither truly individual nor genuine choices at all.

The greatest improvements in child health have therefore often resulted from structural rather than individual interventions. Smoke-free legislation reduced children’s exposure to secondhand smoke and improved perinatal outcomes [9], while the UK Soft Drinks Industry Levy has been associated with reductions in childhood asthma admissions [10]. Rather than expecting individuals to overcome structural disadvantage through behavior change alone, these policies demonstrate that structural change to modify the environments that shape child health delivers improvements across entire populations.

The implications for research and policy are clear. If the ambition is to improve child health and reduce inequalities, then interventions directed solely at changing maternal behaviors are unlikely to achieve substantial population-level gains. Instead, greater attention must be paid to the social and economic conditions in which pregnancies occur. By combining rigorous causal methods with transparent, openly accessible analyses, Sharp and colleagues have provided a model for how epidemiological research can move beyond describing associations to informing meaningful action. Their findings challenge us not only to reconsider where responsibility for child health lies, but also to ensure that future research, public health messaging and policy are guided by robust evidence and focused on addressing the structural determinants that shape child health.

## References

[1] Sharp GC, Lawlor DA, Richardson SS. It’s the mother!: how assumptions about the causal primacy of maternal effects influence research on the developmental origins of health and disease. Soc Sci Med. 2018;213:20–7. doi: 10.1016/j.socscimed.2018.07.035 30055422 PMC6137073

[2] Sharp GC, Lawlor DA, Easey KE, Kundu S, Elhakeem A, Hone E, et al. Exploring parental prenatal influences on child health: a multicohort study and data visualisation tool. PLoS Med. 2026;23(7):e1005153. doi: 10.1371/journal.pmed.1005153 42490625 PMC13395330

[3] Ayorinde A, Esan OB, Buabeng R, Taylor B, Salway S. Ethnic inequities in maternal health. BMJ. 2023;381:1040. doi: 10.1136/bmj.p1040 37172960

[4] Rod NH, Bengtsson J, Budtz-Jørgensen E, Clipet-Jensen C, Taylor-Robinson D, Andersen A-MN, et al. Trajectories of childhood adversity and mortality in early adulthood: a population-based cohort study. Lancet. 2020;396(10249):489–97. doi: 10.1016/S0140-6736(20)30621-8 32798491

[5] Richterman A, Thirumurthy H. Rx Kids: designing cash transfers as health policy. Lancet Public Health. 2026;11(6):e350–1. doi: 10.1016/S2468-2667(26)00094-0 42202815

[6] Lee AR, Kingdon CC, Davie M, Hawcutt D, Sinha IP. Child poverty and health inequalities in the UK: a guide for paediatricians. Arch Dis Child. 2023;108(2):94–101. doi: 10.1136/archdischild-2021-323671 35680401

[7] The Food F. The broken plate. London; 2026. Available from: https://foodfoundation.org.uk/publication/broken-plate-2026

[8] Department for Energy S, Net Z. Fuel Poverty Strategy for England. London; 2026.

[9] Faber T, Kumar A, Mackenbach JP, Millett C, Basu S, Sheikh A, et al. Effect of tobacco control policies on perinatal and child health: a systematic review and meta-analysis. Lancet Public Health. 2017;2(9):e420–37. doi: 10.1016/S2468-2667(17)30144-5 28944313 PMC5592249

[10] Rogers NT, Cummins S, Jones CP, Mytton OT, Roberts CH, Shaheen SO, et al. The UK Soft Drinks Industry Levy and childhood hospital admissions for asthma in England. Nat Commun. 2024;15(1):4934. doi: 10.1038/s41467-024-49120-4 38858369 PMC11164966

